# Network analysis of the relationship between non-suicidal self-injury, depression, and childhood trauma in adolescents

**DOI:** 10.1186/s40359-024-01729-2

**Published:** 2024-04-25

**Authors:** Hui Lei, Yanci Yang, Ting Zhu, Xiaocui Zhang, Junhua Dang

**Affiliations:** 1 College of Education, Hunan Agricultural University, Changsha, Hunan China; 2 Medical Psychological Center, the Second Xiangya Hospital, Central South University, No. 139, Renmin Road, 410011 Changsha, Hunan China; 3 Medical Psychological Institute of Central South University, Changsha, Hunan China; 4 National Clinical Research Center for Mental Disorders, Changsha, Hunan China; 5 Institute of Social Psychology, School of Humanities and Social Sciences, Xi’an Jiaotong University, Xi’an, China

**Keywords:** Adolescent, Non-suicidal self-injury, Depression, Childhood trauma, Network analysis

## Abstract

**Background:**

Non-suicidal self-injury seriously harm the physical and mental health of adolescents. The aim of the current study was to explore the relationship between non-suicide self-injury, depression, and childhood trauma from the perspective of symptoms in adolescents.

**Methods:**

A cross-sectional survey was conducted in four junior high middle schools and collected 2640 valid questionnaires. There were 1329 male students and 1311 female students. The age of the participants ranged from 11 to 17 years old, with a mean age of 13.3 (± 0.94) years. Non-suicidal self-injury (NSSI), depressive symptoms, and childhood trauma were assessed using the Adolescent Self-Harm Scale, the Childhood Depression Scale, and the Childhood Trauma Questionnaire, respectively. A network analysis was performed.

**Results:**

In the network, NSSI, depressive symptoms, and childhood trauma were closely related. Negative self-esteem in the depressive symptoms and emotional abuse in childhood were the most central nodes. Negative self-esteem and negative mood were directly connected to NSSI, other nodes of depressive symptoms appeared to be indirectly connected to NSSI through these two nodes. Emotional abuse was the only node in childhood trauma categories directly connected to NSSI. Nodes of other categories of childhood trauma (physical neglect, physical abuse, emotional neglect, and sexual abuse) were indirectly connected to NSSI through emotional abuse.

**Conclusions:**

NSSI, depression, and childhood trauma of teenagers were closely related. Individuals who have suffered emotional abuse in childhood were more likely to have depressive symptoms and NSSI. Improving negative self-esteem and negative emotions and reducing emotional abuse may be beneficial in alleviating depression and reducing NSSI in adolescents.

**Supplementary Information:**

The online version contains supplementary material available at 10.1186/s40359-024-01729-2.

## Background

Non-suicidal self-injury (NSSI) is the behavior of an individual who intentionally and repeatedly hurt themselves without committing suicide, and such actions are not accepted by social culture [1]. NSSI has become a serious and common problem among adolescents. The detection rate of NSSI in non-clinical adolescents worldwide from 1989 to 2018 was 19.5% [2]. A meta-analysis showed that the detection rate of NSSI in Chinese adolescents was 22.37% [3], and the detection rate of NSSI was higher in adolescent clinical samples [4]. NSSI is often viewed as a way to relieve negative emotions [1], while depression is a common negative emotional experience characterized by significant and persistent mood depression, with symptoms including sadness, anxiety, guilt, attention deficit, etc [5]. A study showed that 24.3% of adolescents suffer from depressive symptoms [6]. Depression and NSSI not only impair adolescents’ current development and adaptation, but also have negative effects that may extend into adulthood, increase the risk of serious psychological disorders, and lead to various behavioral problems, such as suicidal behaviors [7], which seriously harm the physical and mental health of adolescents.

Previous studies have shown a reciprocal relationship between NSSI and depression. Nock and colleagues put forward a four-function model of self-injury, which divided self-injury into four categories according to their functions: individual positive reinforcement, individual negative reinforcement, interpersonal positive reinforcement, and interpersonal negative reinforcement [8]. This model suggests that individual negative reinforcement is the core function of self-injury, such that the main reason of self-injury is to escape from negative emotions. Similarly, Chapman et al.’s experience avoidance model also holds that the function of self-injury is to get rid of the unpleasant emotional experience, and its essence is an emotional avoidance function [9]. When individuals experience negative emotions, they will repeat self-injury because they get instant satisfaction after the first self-injury. Therefore, depression predicts NSSI because individuals will take more self-injurious behavior to cope with the depression to relieve such unpleasant experiences [10–13]. It has also been found that depression is a consequence of self-injury [14, 15]. Individuals exhibit self-injurious behavior due to certain factors, which in turn leads to negative emotional experiences such as guilt and thus exacerbates depressive symptoms. Therefore, NSSI also predicts depression.

Previous studies have found that multiple factors could predict depression and NSSI, such as genetics, childhood trauma, and parental conflict [16–21]. Individuals who have experienced childhood trauma tend to have more depressive symptoms [22] and a higher tendency of self-injury [23]. Particularly, research shows that different dimensions of childhood trauma are disproportionately associated with individual mental health. For example, it has been found that individuals who had experienced emotional abuse [24] or sexual abuse [25] were more prone to NSSI. Wang et al. measured childhood trauma in young people with depression and found that high levels of emotional neglect and emotional abuse were associated with anhedonia, an important feature of depression [26]. Meanwhile, recent studies showed that different symptoms of depression may also have different degrees of association with NSSI [27]. Therefore, we need to further explore which symptoms of depression are directly related to NSSI so that we can take prompt action for prevention when the corresponding symptoms appear.

The current study aims to utilize network analysis to reveal the complex relationships among NSSI, different symptoms of depression, and various dimensions of childhood trauma. The network analysis approach is to find the more central or important variables in the network by using nodes to represent the observed variables and using lines to represent the correlation between the observed variables under the drive of data [28]. A node with high centrality has the characteristic that when it is activated, it is likely to spread the activation to the entire network through the edges connecting to other nodes, thus affecting the entire symptom network [29]. That is, if an intervention is performed on nodes with high cardinality, it may reduce the severity of the entire symptom network. Network analysis can also describe the complex relationships between observed variables, such as the co-morbidity between obsessive-compulsive disorder and depression [30], and the relationships among eating disorders, self-esteem, and depression in adolescents [31]. More relevant to the current study, Misiak et al. [32] explored the relationships between childhood trauma, psychotic-like experiences, depression, and NSSI in a 18-35-year-old group with a negative history of psychiatric treatment using network analysis. It was found that sexual abuse and longer lifetime duration of NSSI were the most central nodes in the network, but the associations of childhood trauma and psychotic-like experiences with NSSI might be independent. However, these results may not be generalized to a non-clinical adolescent sample.

Therefore, this study intends to investigate the complex relationships among adolescents’ NSSI, depression, and childhood trauma utilizing network analysis in a non-clinical adolescent sample, with a particular focus on the indicators of high central nodes in the network, to provide visualization results for understanding the relationships among these variables and provide specific directions for future interventions. Based on previous studies reviewed above, emotional abuse may be particularly important because it has been linked with both depression [26] and NSSI [24].

## Methods

### Settings and participants

A cluster sampling method was adopted to investigate four junior middle schools in Zhangjiajie, China, and questionnaires were administered to students in class units. In this study, 2870 questionnaires were handed out, and 2640 valid questionnaires were returned, including 1329 male students (50.3%) and 1311 female students (49.7%), and 974 students (36.9%) in the first grade, 863 students (32.7%) in the second grade, and 803 students (30.4%) in the third grade. There are 497 boys and 477 girls in the first grade, 440 boys and 423 girls in the second grade, 392 boys and 411girls in the third grade. The age of the participants ranged from 11 to 17 years old, with a mean age of 13.3 (± 0.94) years. All participants and their guardians provided written informed consent. The questionnaire was distributed by psychology students and accompanied by the class teachers. After the test, teachers specialized in mental health, who were mandatorily manned in each school, carried out theme activities to alleviate the negative emotions that students may have in answering the questionnaire. The teachers educated the students about the importance of mental health, the detriment and inappropriateness of NSSI, the right ways of coping with stress and negative emotions, and how to report to the schools when they experience abuse and neglect. This study was conducted in compliance with the Declaration of Helsinki and received ethical approval from the Ethics Committee of Hunan Agricultural University.

### Measurements

#### The adolescent Self-Injury Scale (ASIS)

The ASIS was revised by Jiang et al. [33] to evaluate the frequency and severity of NSSI behavior adolescents experienced in their past lifetimes. It includes 18 items indicating 18 common ways of NSSI in Chinese adolescents (such as stabbing, scratching and burning). Each item was evaluated from two dimensions: frequency and severity. The frequency was divided into four levels: 0 times, 1 time, 2–4 times, and more than 5 times (including 5 times). The severity was coded into a 5-level scale from 0 to 4 (0-none, 1-mild, 2-moderate, 3-severe, 4-extremely severe). The score of a single item is the product of its frequency and severity. The total score of the ASIS was the sum of the 18 items, which represents the overall severity of NSSI. The questionnaire has good internal consistency coefficients and criterion correlation validity and most Chinese researchers have used this questionnaire in their self-injurious behavior surveys [34, 35]. The Cronbach’s alpha coefficient of this scale in our sample was 0.94.

#### The Childhood Depression Scale (CDI)

The CDI was used to assess depressive symptoms in children and adolescents. The scale was developed by Kovacs [36] and translated by Wu et al. [37]. Wu et al. [37] analyzed the reliability and validity of the Chinese version in middle school students and found that the CDI scale was suitable for the assessment of depressive symptoms in Chinese middle school students. The scale applies to ages 7–17 and has 27 items divided into five subscales: anhedonia, negative emotions, negative self-esteem, ineffectiveness, and interpersonal problems. Each item has three options describing different degrees of depressive symptoms, which are calculated as 0 to 2 points, in which 0 means the least degree of depressive symptoms, followed by 1, and 2 means the most severe degree of depressive symptoms. The total score on the scale is 54 points, with higher scores indicating greater depression. The sum score of 20 was identified as the optimal screening cut-off score [36]. The Cronbach’s alpha coefficient of the CDI was 0.90 in our sample.

#### The Childhood Trauma Questionnaire-Short Form (CTQ-SF)

The CTQ-SF, developed by Bernstein et al. [38], and translated by Zhao et al. [39], was used for the measurement of childhood trauma. A total of 28 questions are included in the CTQ-SF, each rated on a five-point scale: never = 1, occasionally = 2, sometimes = 3, often = 4, and always = 5, with a total score range of 28 to 140. The questionnaire contains 5 types of trauma assessment: physical abuse (PA, defined as, “bodily assaults on a child by an adult or older person that posed a risk of or resulted in injury.”), physical neglect (PN, defined as, “the failure of caretakers to provide for a child’s basic physical needs, including food, shelter, clothing, safety, and health care”), emotional neglect (EN, defined as, “the failure of caretakers to meet children’s basic emotional and psychological needs, including love, belonging, nurturance, and support.”), emotional abuse (EA, defined as, “verbal assaults on a child’s sense of worth or well-being or any humiliating or demeaning behavior directed toward a child by an adult or older person.”), and sexual abuse (SA, defined as “sexual contact or conduct between a child younger than 18 years of age and an adult or older person.”). In the subscale, emotional abuse ≥ 13 points, physical abuse ≥ 10 points, sexual abuse ≥ 8 points, emotional neglect ≥ 15 points and physical neglect ≥ 10 points are defined as childhood trauma of corresponding dimensions [40]. The scores for each childhood trauma type range from 5 to 25. Higher scores on the abuse and neglect subscales reflect greater severity of childhood traumatic experiences. Studies have confirmed that CTQ-SF is an effective and reliable tool to evaluate experiences of child abuse in China [41]. The Cronbach’s alpha of this scale was 0.72 in our sample.

### Network estimation

Statistical analyses were performed using SPSS24.0 and R Studio software. Descriptive statistics were first performed on the sample. The relationship between depression, self-injury, and childhood trauma in adolescents was studied by network analysis.

Network analysis is a data-driven analysis method that allows exploring not only the independent relationship between every two variables but also the independent role of multiple variables in a complex system [42]. Firstly, the *qgraph* package of R studio software [43] was used to establish a partial correlation network model among adolescent NSSI, five factors of depression, and five factors of childhood trauma. The network structure includes nodes and edges. The nodes represent the observed variables, and the edges represent the correlation coefficients between two nodes after controlling for all other variables [44]. The strength of the relationship between two nodes is presented by the thickness of the connected edge. The stronger the relationship between two nodes, the thicker the connected edge. Positive correlations are shown by solid green lines, while negative correlations are shown by dotted red lines. The partial correlation coefficient between the nodes is calculated using the *ppcor* package of R studio software. Next, the importance of each node in the network is calculated, i.e., the importance of each node in the network is measured by node strength [45]. We usually consider the node with the highest node strength as the most important and central node in the whole network. Finally, we use the *bootnet* package of R studio software to assess the stability of the network. The stability of node centrality is evaluated by calculating the correlation stability (CS) coefficient through the sample descent self-help method. Previous studies have shown that the CS coefficient ≥ 0.5 indicates sufficient stability [46]. The R code for the network analyses are reported in Supplementary materials “R code.R”.

Eleven nodes are included in this study: anhedonia (Anh), negative mood (NgM), negative self-esteem (NSE), ineffectiveness (Inf), interpersonal Problem (InP), ASIS (ASI), physical neglect (PN), physical abuse (PA), emotional neglect (EN), emotional abuse (EA), and sexual abuse (SA).

## Results

In our study, the lifetime prevalence of NSSI was 41% and the detection rate of depressive symptoms was 27%. For the prevalence of childhood trauma, 253 teenagers experienced emotional abuse, accounting for 9.5%; 67 teenagers experienced physical abuse, accounting for 2.5%; 49 teenagers experienced sexual abuse, accounting for 1.8%; 1034 teenagers experienced emotional neglect, accounting for 39%; and 380 teenagers experienced physical neglect, accounting for 14%.

**Fig. 1:**
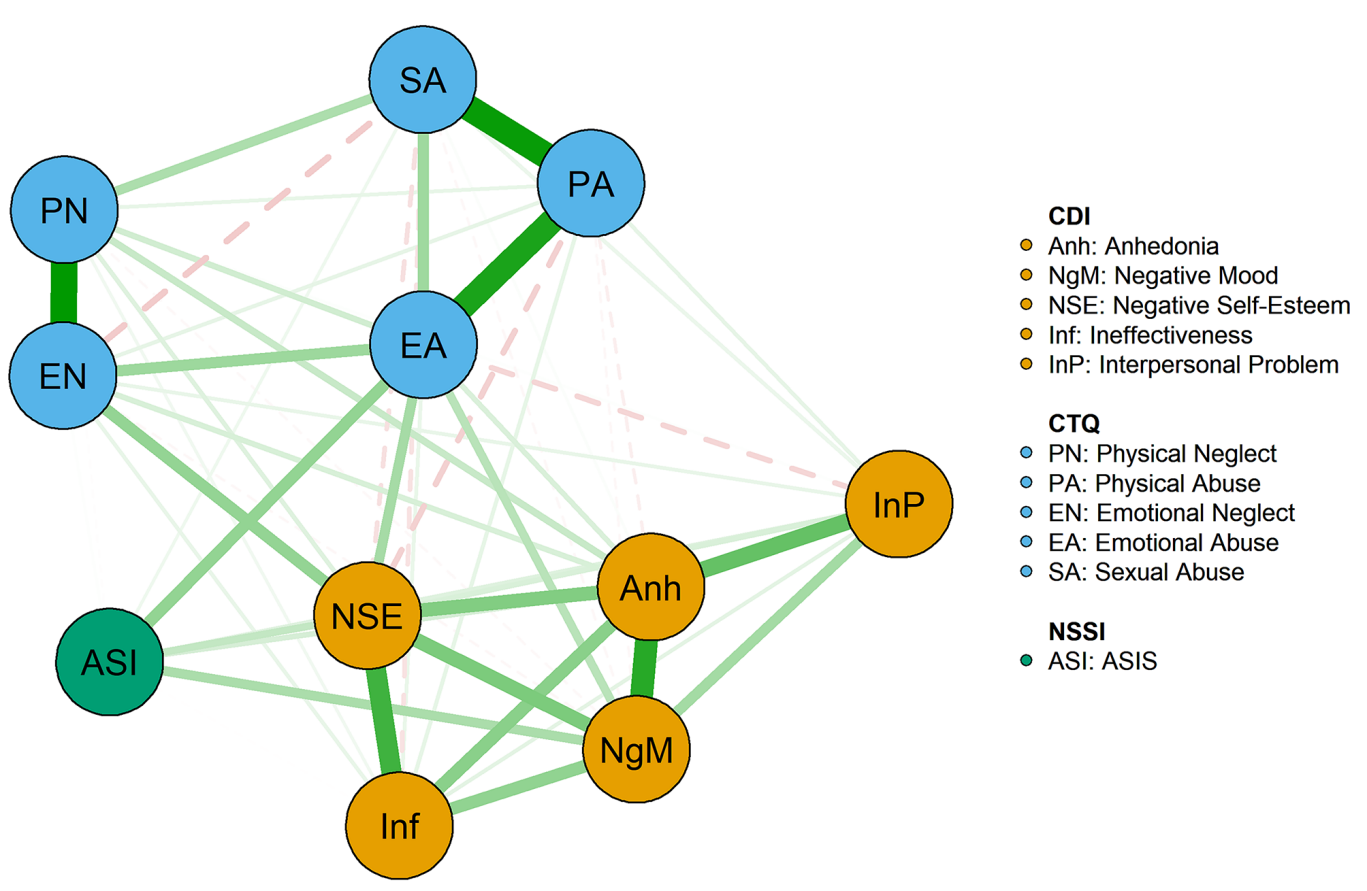
Symptom networks of adolescent non-suicidal self-injury, depression, and childhood trauma. In the figure, green nodes represent NSSI; blue nodes represent depressive symptoms, and orange nodes represent different types of childhood trauma. The solid green line represents positive correlations; the red dashed line represents negative correlations. The edge thickness of the line represents the strength of the association between symptom nodes

The biased correlation network model was shown in Fig. 1; Table 1. Among the five factors of adolescent depression, the strongest linkage weights were anhedonia and negative mood (*r* = 0.32); Among the five factors of childhood trauma, physical neglect and emotional neglect (*r* = 0.38) had the strongest wired weight. The strongest weight of the linkage between NSSI and depressive symptoms were ASIS (NSSI) and negative mood (*r* = 0.13); while ASIS (NSSI) and emotional abuse (*r* = 0.16) were the strongest weight of the linkage between NSSI and childhood trauma. The weights of these edges did differ significantly (Supplementary Figure S1).

**Table 1:** Weight of connections of each node in the network

	M	SD	Anhedonia	Negative Mood	Negative Self Esteem	Ineffectiveness	Interpersonal Problem	ASIS	Physical Neglect	Physical Abuse	Emotional Neglect	Emotional Abuse	Sexual Abuse
Anhedonia	3.97	2.97	0										
Negative Mood	2.70	2.22	0.32	0									
Negative Self Esteem	2.56	1.90	0.19	0.20	0								
Ineffectiveness	2.88	1.89	0.18	0.17	0.28	0							
Interpersonal Problem	1.65	1.26	0.23	0.14	0.06	0.04	0						
ASIS	5.28	13.16	0.06	0.13	0.09	0.00	0.05	0					
Physical Neglect	9.18	3.00	0.08	-0.01	0.05	0.03	0.01	0.00	0				
Physical Abuse	6.04	2.24	-0.03	-0.01	-0.06	0.04	0.03	0.04	0.04	0			
Emotional Neglect	11.91	4.77	0.06	-0.01	0.16	0.03	0.03	0.01	0.38	0.03	0		
Emotional Abuse	7.77	3.30	0.06	0.10	0.14	0.03	-0.06	0.16	0.07	0.34	0.15	0	
Sexual Abuse	5.55	1.91	0.01	-0.02	-0.05	-0.04	0.04	0.03	0.12	0.38	-0.07	0.14	0

As shown in Fig. 1, negative self-esteem and negative mood were directly connected to NSSI, other nodes of depressive symptoms appeared to be indirectly connected to NSSI. For example, node ineffectiveness itself did not affect the NSSI, but it worked through the node negative self-esteem. Similarly, the node emotional abuse was the only node in the childhood trauma categories directly connected to NSSI. Nodes of other categories of childhood trauma (physical neglect, physical abuse, emotional neglect, and sexual abuse) were indirectly connected to NSSI through emotional abuse.

**Fig. 2:**
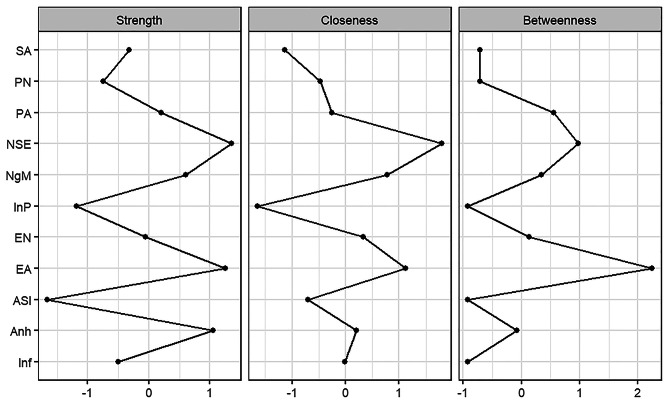
Centrality measures of network nodes. The figure shows the indicators of centrality (i.e., strength, tightness, and mediation) of all node symptoms in the network. Note: Anh (Anhedonia), NgM (Negative Mood), NSE (Negative Self Esteem), Inf (Ineffectiveness), InP (Interpersonal Problem), ASI (ASIS), PN (Physical Neglect), PA (Physical Abuse), EN (Emotional Neglect), EA (Emotional Abuse), SA (Sexual Abuse)

Centrality indicators for the network were presented in Fig. 2. For the depressive symptoms, node negative self-esteem had the greatest degree of direct connection with other nodes (i.e., having the highest level of strength) and the greatest indirect connection with other nodes in the network (i.e., the highest level of closeness). Also, node negative self-esteem was directly or indirectly involved in processing of the network during the interaction of variables (i.e., the highest level of betweenness). For the childhood trauma, node emotional abuse showed the highest levels of strength, closeness, and betweenness centrality indices. Results of the analysis for between-node differences in the strength centrality index, betweenness centrality index and closeness centrality index are illustrated in Supplementary Figure S2-S4.

**Fig. 3:**
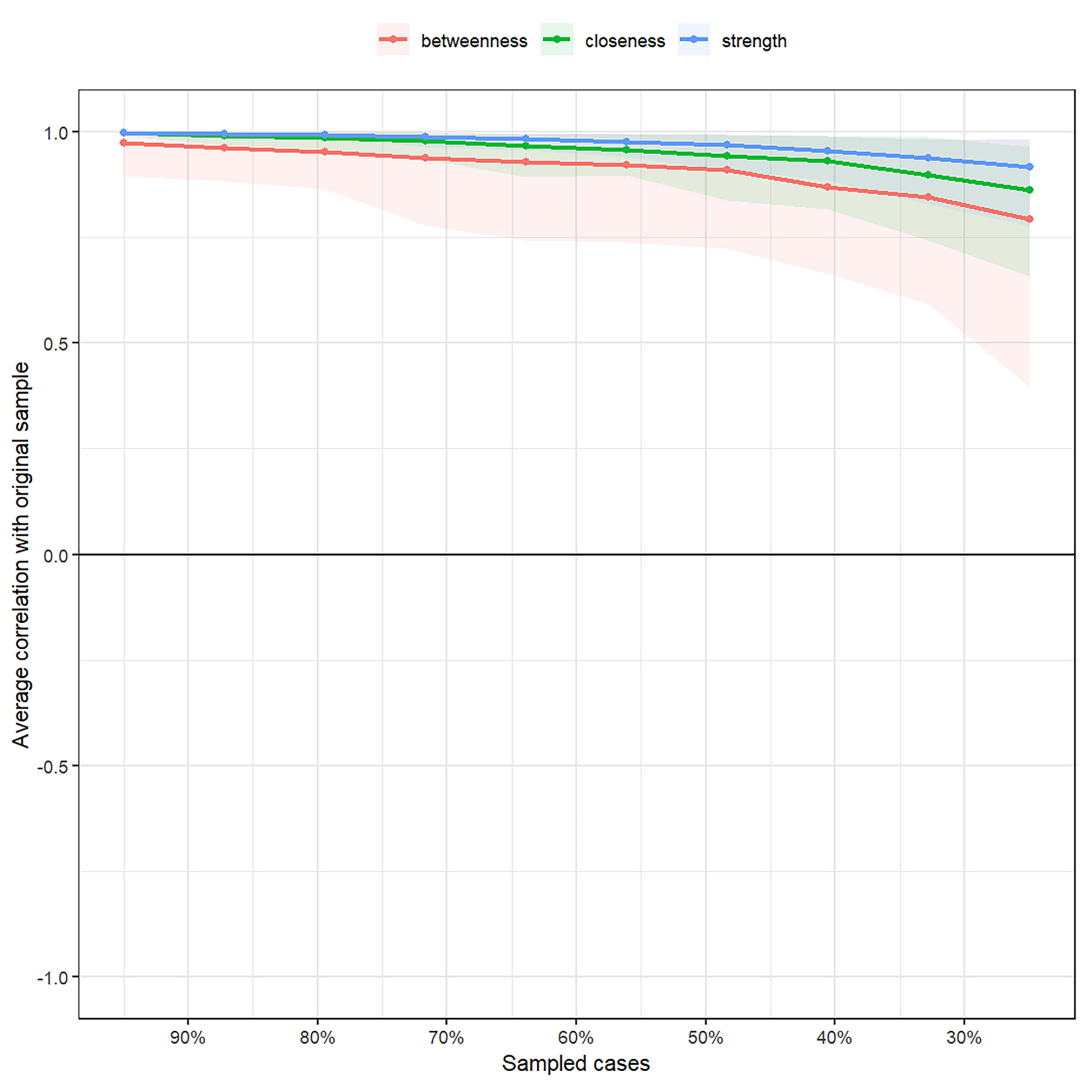
Stability detection of network centrality measures. The blue line indicates the stability of node strength, the green line indicates the stability of node tightness, the red line indicates the stability of node mediation, and the area indicates the 95% confidence interval

Further stability tests of the network centrality measures found (Fig. 3) that the correlation stability (CS) coefficients of node strength, node tightness, and node intermediation were 0.75, 0.67, and 0.59, respectively, with node strength having the best stability and CS coefficients all greater than 0.5, indicating that all the node centrality measures of this network have good stability.

## Discussion

In this study, the relationship between NSSI, depression, and childhood trauma was explored using network analysis, and the important nodes in the network were analyzed. NSSI, depressive symptoms, and childhood trauma were closely linked in the network, suggesting that the symptoms have an interactive influence on each other rather than being independent of each other. First, there is a close relationship between childhood trauma and NSSI and depression. Previous studies reported that childhood trauma can predict depression [47] and NSSI [48, 49]. The results of our study confirm the idea proposed by Eisenberg’s the socialization of emotion theory [50]: if parents or caregivers treat their children with forms of indifference, neglect, or abuse, the children will lack the necessary sense of belonging and security, and then they might not be able to adjust their emotions, leading to the emergence of depressive symptoms. From the theory and our results, they will take unique ways to relieve the current uncomfortable feelings, such as NSSI, to protect themselves.

Second, different childhood traumatic experiences have different effects on depression and NSSI, which is consistent with previous studies. Heleniak et al. [51] found a strong association between childhood pleasure deficit mood disorder and child maltreatment in the sample of community adolescents; Zhang et al. [24] found that the risk factor for NSSI was emotional abuse. Finally, there is a reciprocal predictive relationship between NSSI and depression in adolescents [52]. The present study further found that adolescent NSSI was more strongly linked to the depression network and relatively more weakly linked to the childhood trauma network. This may be due to the early occurrence of childhood trauma, and the effect on adolescent NSSI behavior may be weakened with the passage of time. However, depression, as a persistent negative emotion, has a more immediate and significant impact on adolescents’ NSSI behavior.

In the depressive symptoms of adolescents, negative self-esteem had the strongest node-centrality. The cognitive susceptibility model of depression suggests that when individuals hold negative self-perception, they are more likely to adopt a negative attitude to anticipate the future, resulting in depression [53]. Low self-esteem is a cognitive tendency like negative self-evaluation, which has a great impact on the occurrence and maintenance of depression [54].

Emotional abuse was the strongest centrality node in the childhood trauma, which was closely related to both physical abuse and sexual abuse. Emotional abuse may destroy an individual’s ability to regulate negative emotions. Individuals who experience emotional abuse tend to adopt dysfunctional coping mechanisms, such as NSSI, to relieve painful emotions if they experience other types of abuse again in the absence of positive resilience to negative emotions [55, 56]. This finding is different from a recent study [32]. Misiak et al. found that a history of childhood sexual abuse was the most central node in the network and other types of childhood trauma were connected to the lifetime characteristics through sexual abuse. It may be caused by regional or cultural differences. Our findings are also different from the traditional parents’ or caretakers’ thoughts in China. Parents or caretakers in China may think that physical abuse such as corporal punishment is the culprit of childhood trauma, so they commonly choose negative words that seem harmless to educate their children, but this behavior eventually leads to more serious emotional abuse of children. Our results also suggest that emotional abuse like physical abuse will do indelible harm to children, so parents or caretakers should not ignore the negative effects of emotional abuse on children’s physical and mental development.

We also found that emotional abuse was a very important node in the network that connected adolescent NSSI, depression, childhood trauma. Firstly, other childhood traumas established contact with NSSI through it. This indicates that emotional abuse and other types of abuse will continuously increase emotional abuse behaviors and lead to NSSI. Secondly, emotional abuse can lead to depressive symptoms such as negative self-esteem and negative mood to indirectly induce NSSI, so we need to pay particular attention to children who have suffered emotional abuse. Heleniak et al. [51] also found in their study of the relationship between psychological abuse and children’s pleasure deficits that psychological abuse and children’s pleasure deficits were positively correlated, suggesting that emotional abuse can, to some extent predict the production of depression.

In the same way, negative self-esteem and negative mood were also two important nodes in the network, which were not only important central nodes in the depressive symptoms but also played a bridging role in the whole network, i.e., other depressive symptoms acted on NSSI through these two symptoms. Therefore, the intervention for NSSI needs to focus on these two depressive symptoms. Network analysis is important for more targeted prevention of adolescent NSSI behaviors, and can play its role in the practical application of psychotherapy. When important nodal symptoms appear in the network, we can quickly take measures to intervene in other nodal symptoms that are most closely linked to them and achieve prevention of the emergence of psychological disorders or risky behaviors. For example, in this study, node emotional abuse is most closely associated with nodes negative self-esteem, negative mood, and NSSI, so when dealing with adolescents who have experienced childhood emotional abuse, it is important to focus on whether they show low self-esteem and negative emotions to help us make a quick diagnosis or intervention.

Combined with our research, it is found that improving teenagers’ self-esteem and being alert to the harm of emotion abuse will help reduce the incidence of non-suicidal self-injury behavior of teenagers and effectively alleviate their depression. Through the cooperation of schools, families and communities, caretakers can be made aware of the harm of emotional abuse and adopt gentle and supportive parenting methods. Schools can improve the positive psychological quality of teenagers through relevant education and promote their physical and mental health development.

The current study has some limitations. First, this study used cross-sectional data and the assessment tools have various time periods, which do not allow us to infer much on causality. To describe the directivity of the relationship, we can use the network analysis method to analyze the longitudinal data and explore the temporal causality between nodes in future studies. Second, the questionnaires of the study were all retrospective, and recall bias could not be avoided. Third, the survey population of this study was limited to China, and it is unclear whether the results are applicable to teenagers from other countries or different cultural backgrounds. Fourth, our sample may include adolescents with mental problems or mental disorders, and subsequent studies can further screen and investigate whether the current results can also be applied to them. Finally, we only consider the prevalence of NSSI, and future research can focus on more specific effects of NSSI behavior.

## Conclusions

In conclusions, we used the network analysis to explore the relationship between NSSI, depression, and childhood trauma in adolescents, and found that adolescent NSSI, depression, and childhood trauma interacted with each other. The two depressive symptoms, including negative self-esteem and negative mood, and emotional abuse were important node. These findings indicate that NSSI, depression and childhood trauma of teenagers were closely related. Individuals who have suffered emotional abuse in childhood were more likely to have depressive symptoms and NSSI. Improving negative self-esteem and negative emotions and reducing emotional abuse may go a long way in alleviating depression and reducing NSSI in adolescents.

### Electronic supplementary material

Below is the link to the electronic supplementary material.

Supplementary Material 1

Supplementary Material 2

## Data Availability

The datasets supporting this study’s findings are available from the corresponding author upon reasonable request.

## Abbreviations

NSSI: Non-suicidal self-injury
