# Supplementary material for: Network analysis of the relationship between non-suicidal self-injury, depression, and childhood trauma in adolescents
Source: BMC Psychol. 2024 Apr 25;12:234. doi: 10.1186/s40359-024-01729-2 (PMC11046936; doi:10.1186/s40359-024-01729-2)
Supplement: Supplementary file 2 — Supplementary Material 2 [file 40359_2024_1729_MOESM2_ESM.docx]

**Supplementary Figure Legends**

**Figure S1.** Bootstrapped differences between edge weights. Significant differences between edge weights are marked as black boxes.

**Figure S2.** Bootstrapped differences between node strengths. Significant differences are marked as black boxes.

**Figure S3.** Bootstrapped differences between node betweennesses. Significant differences are marked as black boxes.

**Figure S4.** Bootstrapped differences between node closenesses. Significant differences are marked as black boxes.

**Figure S1**

**
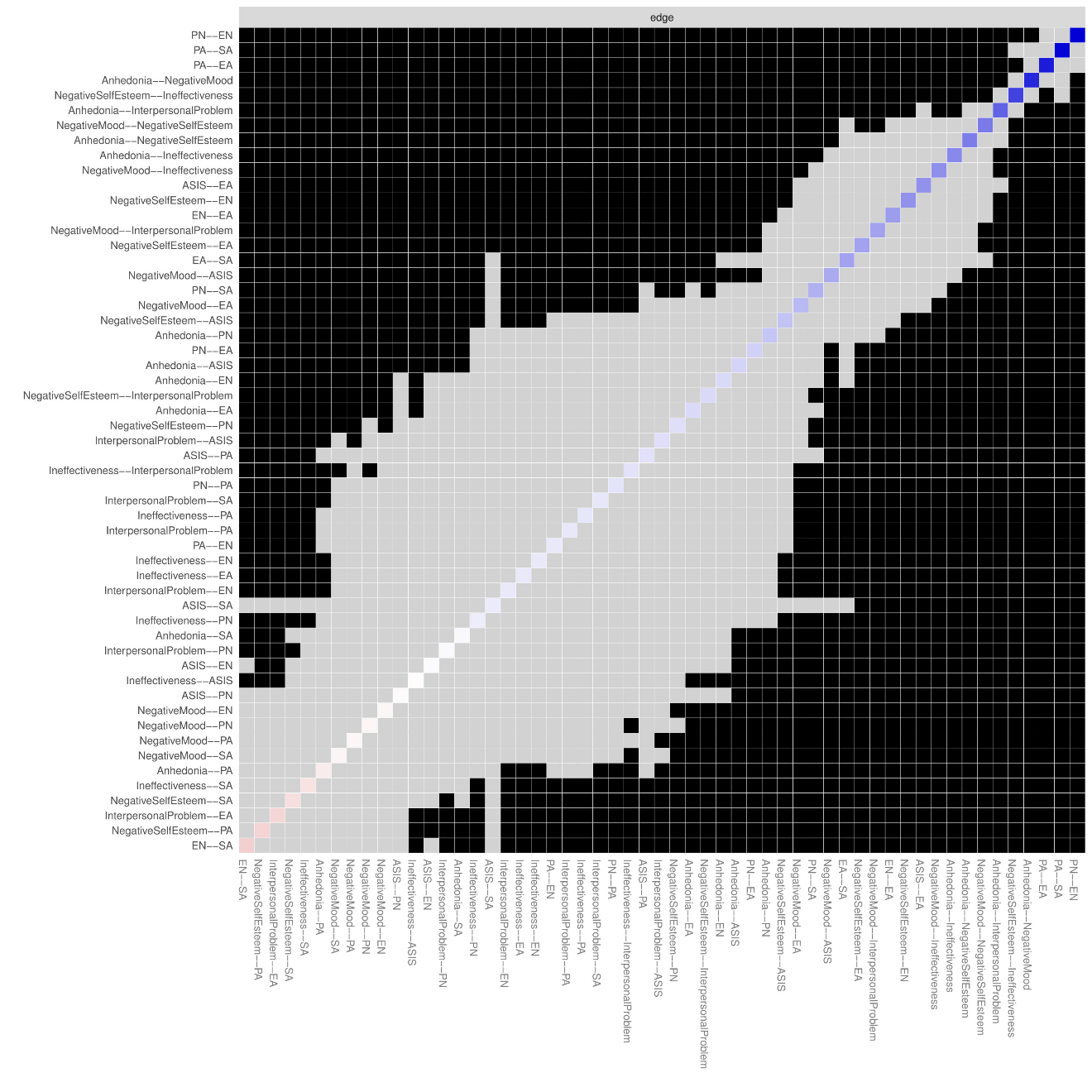
**

**Figure S2**

**
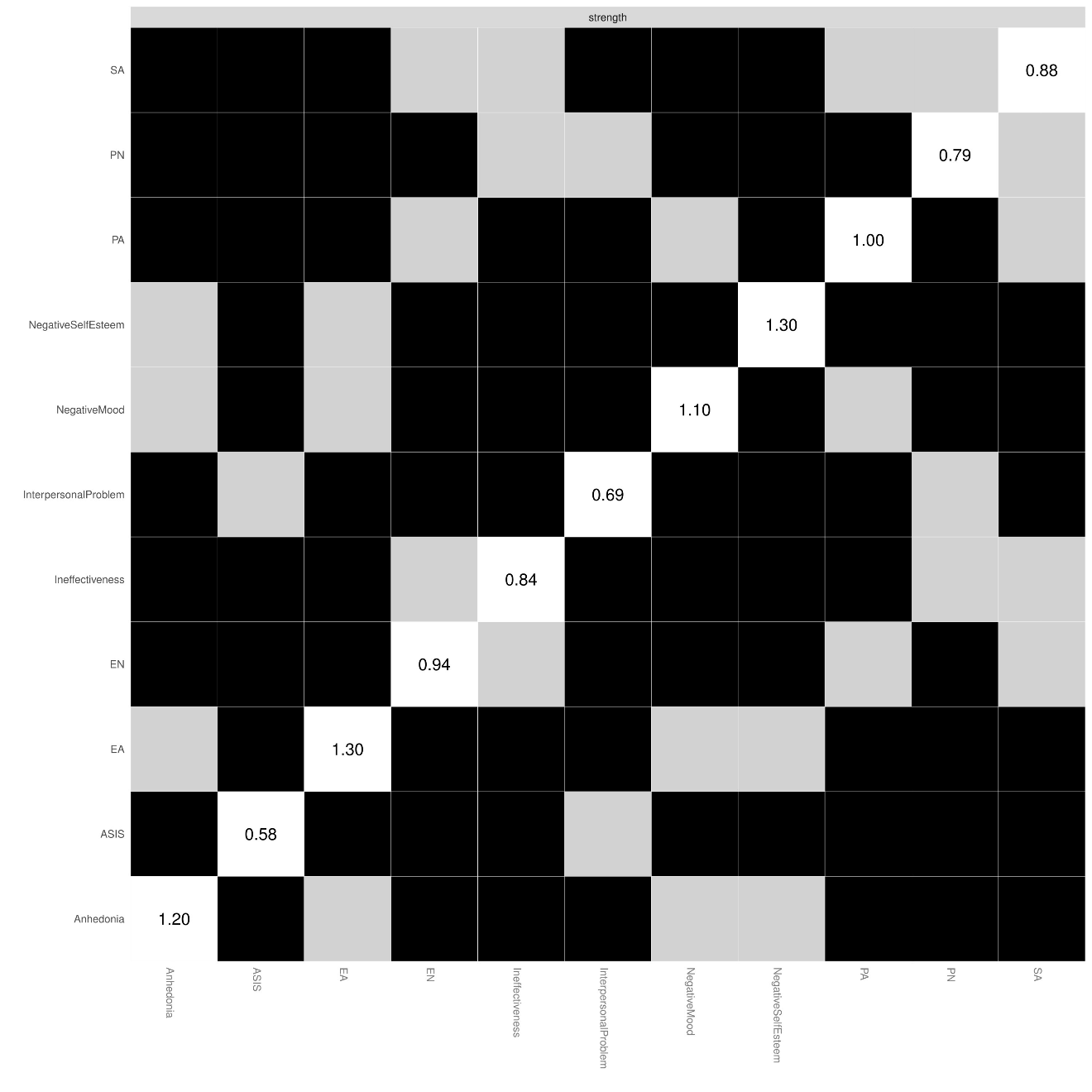
**

**Figure S3**

**
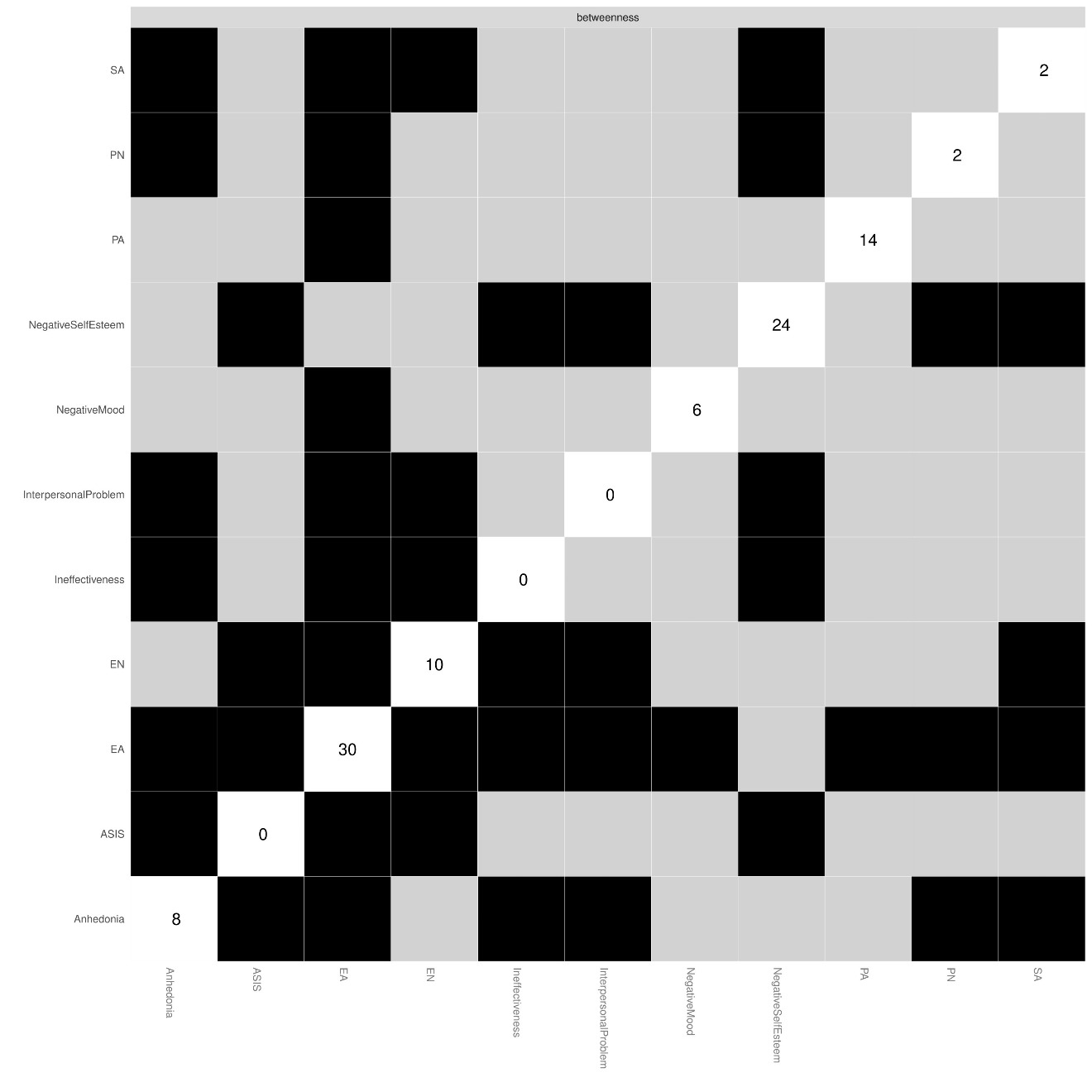
**

**Figure S4**

**
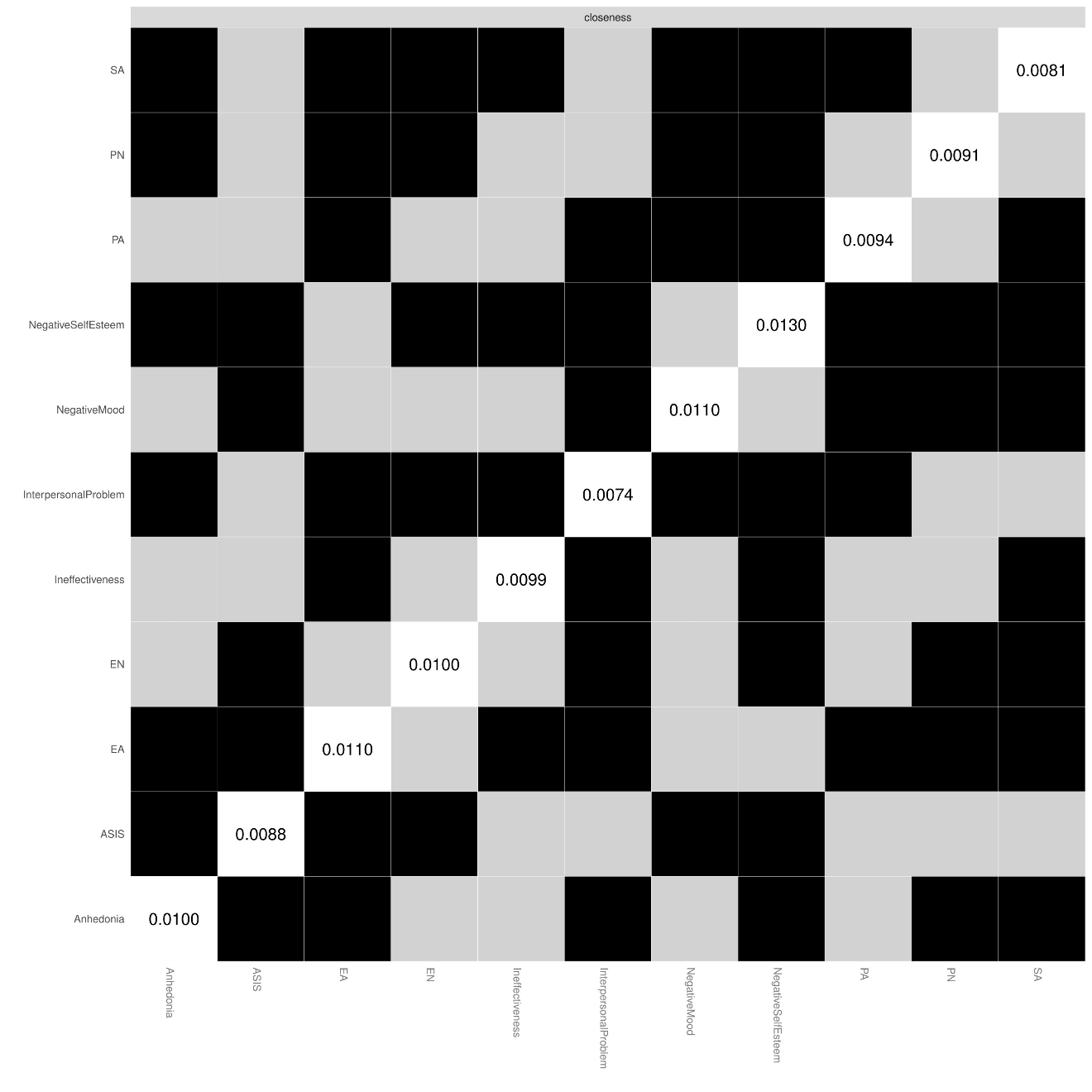
**
